# Monodeuterated Methane, an Isotopic Tool To Assess Biological Methane Metabolism Rates

**DOI:** 10.1128/mSphereDirect.00309-17

**Published:** 2017-08-23

**Authors:** Jeffrey J. Marlow, Joshua A. Steele, Wiebke Ziebis, Silvan Scheller, David Case, Linda M. Reynard, Victoria J. Orphan

**Affiliations:** a Division of Geological and Planetary Sciences, California Institute of Technology, Pasadena, California, USA; b Department of Biological Science, University of Southern California, Los Angeles, California, USA; c Department of Human Evolutionary Biology, Harvard University, Cambridge, Massachusetts, USA; DOE Joint Genome Institute; Wetsus, European Centre of Excellence for Sustainable Water Technology; University of California, Los Angeles

**Keywords:** environmental microbiology, metabolic rate measurement, methane, stable isotope probing

## Abstract

Biological methane oxidation is a globally relevant process that mediates the flux of an important greenhouse gas through both aerobic and anaerobic metabolic pathways. However, measuring these metabolic rates presents many obstacles, from logistical barriers to regulatory hurdles and poor precision. Here we present a new approach for investigating microbial methane metabolism based on hydrogen atom dynamics, which is complementary to carbon-focused assessments of methanotrophy. The method uses monodeuterated methane (CH_3_D) as a metabolic substrate, quantifying the aqueous D/H ratio over time using off-axis integrated cavity output spectroscopy. This approach represents a nontoxic, comparatively rapid, and straightforward approach that supplements existing radiotopic and stable carbon isotopic methods; by probing hydrogen atoms, it offers an additional dimension for examining rates and pathways of methane metabolism. We provide direct comparisons between the CH_3_D procedure and the well-established ^14^CH_4_ radiotracer method for several methanotrophic systems, including type I and II aerobic methanotroph cultures and methane-seep sediment slurries and carbonate rocks under anoxic and oxic incubation conditions. In all applications tested, methane consumption values calculated via the CH_3_D method were directly and consistently proportional to ^14^C radiolabel-derived methane oxidation rates. We also employed this method in a nontraditional experimental setup, using flexible, gas-impermeable bags to investigate the role of pressure on seep sediment methane oxidation rates. Results revealed an 80% increase over atmospheric pressure in methanotrophic rates the equivalent of ~900-m water depth, highlighting the importance of this parameter on methane metabolism and exhibiting the flexibility of the newly described method.

**IMPORTANCE** Microbial methane consumption is a critical component of the global carbon cycle, with wide-ranging implications for climate regulation and hydrocarbon exploitation. Nonetheless, quantifying methane metabolism typically involves logistically challenging methods and/or specialized equipment; these impediments have limited our understanding of methane fluxes and reservoirs in natural systems, making effective management difficult. Here, we offer an easily implementable, precise method using monodeuterated methane (CH_3_D) that advances three specific aims. First, it allows users to directly compare methane consumption rates between different experimental treatments of the same inoculum. Second, by empirically linking the CH_3_D procedure with the well-established ^14^C radiocarbon approach, we determine absolute scaling factors that facilitate rate measurements for several aerobic and anaerobic systems of interest. Third, CH_3_D represents a helpful tool in evaluating the relationship between methane activation and full oxidation in methanotrophic metabolisms. The procedural advantages, consistency, and novel research questions enabled by the CH_3_D method should prove useful in a wide range of culture-based and environmental microbial systems to further elucidate methane metabolism dynamics.

## Abstract (precis)

Microbial methane consumption is a critical component of the global carbon cycle, with wide-ranging implications for climate regulation and hydrocarbon exploitation. Nonetheless, quantifying methane metabolism typically involves logistically challenging methods and/or specialized equipment; these impediments have limited our understanding of methane fluxes and reservoirs in natural systems, making effective management difficult. Here, we offer an easily implementable, precise method using monodeuterated methane (CH_3_D) that advances three specific aims. First, it allows users to directly compare methane consumption rates between different experimental treatments of the same inoculum. Second, by empirically linking the CH_3_D procedure with the well-established ^14^C radiocarbon approach, we determine absolute scaling factors that facilitate rate measurements for several aerobic and anaerobic systems of interest. Third, CH_3_D represents a helpful tool in evaluating the relationship between methane activation and full oxidation in methanotrophic metabolisms. The procedural advantages, consistency, and novel research questions enabled by the CH_3_D method should prove useful in a wide range of culture-based and environmental microbial systems to further elucidate methane metabolism dynamics.

## INTRODUCTION

Methane-consuming microbial processes represent an important component of biogeochemical cycles in natural freshwater and marine environments, as well as in human-impacted systems. In terrestrial soils, methane production in rice fields, anoxic wetlands, and thawing permafrost supports methanotrophic communities (1–4). In marine settings, an estimated 85 Tg of methane per year, derived from biogenic and thermogenic sources, enters the subseafloor, the vast majority of which is anaerobically consumed in anoxic sediments (5). Much of what remains is taken up in microoxic or oxic zones of the sediment or water column by aerobic methanotrophic microorganisms (6). Methanotrophy is also of interest in a range of human-impacted contexts, including groundwater (7, 8), wastewater treatment plants (9), landfills (10), shale gas (11), coalbed harvesting (12), and oil spills (13).

In addition to its climatic and economic implications, the biochemical details of the methanotrophic process have stimulated many investigations. The sulfate-linked anaerobic oxidation of methane (AOM; reaction 1) has proven particularly enigmatic; this process typically involves a mutualistic relationship between anaerobic methanotrophic (ANME) archaea and sulfate-reducing bacteria (SRB) (14–16), although nitrate (17, 18) and, potentially, metals such as iron and manganese (19–21) can serve as alternative electron acceptors for some ANME lineages. Methane is oxidized aerobically (reaction 2) by members of the classes *Gammaproteobacteria* (e.g., type I and type X) and *Alphaproteobacteria* (type II); verrucomicrobial representatives can perform aerobic methanotrophy under extremely acidic conditions (22, 23). Methane is converted to methanol, which is further oxidized to formaldehyde; assimilatory pathways branching at this point can incorporate carbon into central metabolism through the ribulose monophosphate (RuMP) cycle (type I and type X methanotrophs) or the serine cycle (type II).

(reaction 1) CH2+SO42− →HCO3−+HS−+H2O

(reaction 2) $${\text{CH}}_{4}+2{\text{O}}_{2}\;\to {{\mathrm{HCO}}_{3}}^{-}+{\text{H}}_{2}\text{O}+{\text{H}}^{+}$$

Methanotrophy is both a biogeochemically relevant activity that modulates the global climate and a poorly understood biochemical process; given this dual role, there is substantial interest in measuring its rate and in understanding elemental flows through metabolic pathways. The oxidation of methane in environmental samples has traditionally been studied using a few techniques. Numerical models incorporating environmental sediment profiles of sulfate and methane concentrations can be used to back-calculate methane consumption rates (24). ^13^CH_4_ can be used to probe rates under controlled conditions (25–28), but the presence of natural ^13^C in marine dissolved inorganic carbon (DIC) pools requires long incubations as well as accurate measurements of isotopically resolved concentrations of reactants and products (29). Gas chromatography (GC) quantification of dissolved (30–32) or headspace (33, 34) methane concentrations has also been demonstrated as a rate measurement tool, though low concentrations can hamper reproducibility and exacerbate background contamination issues, particularly in field-based settings (35). Perhaps the most sensitive approach uses radiolabeled ^14^CH_4_ to track the oxidation of methane-associated carbon to inorganic carbon species (36, 37). Tritiated methane was introduced for water column aerobic methane oxidation measurements due to its higher activity per radionuclide (6, 38). Logistical challenges and health and safety regulations led Pack et al. (29) to develop an accelerator mass spectrometry detection method that requires 10^3^ to 10^5^ less radiolabel than previous ^14^C and ^3^H approaches, though the analytical procedure remains labor-intensive.

Despite the range of methods available, measurement of microbial methane utilization rates remains cumbersome, and a precise, safe, and easily enacted approach would be a welcome contribution for a diverse array of researchers. Nearly all of the aforementioned approaches are carbon based; a hydrogen-based tracer offers a complementary approach to investigations of methane biochemical dynamics. Here we introduce a novel method for biologically mediated methanotrophy rate measurement that utilizes monodeuterated methane (CH_3_D) as a substrate and measures the D/H ratio of the aqueous solution. This approach offers several advantages for prospective users: it does not require the logistical, safety, and administrative hurdles associated with radiotracers such as ^14^CH_4_ and [^3^H]CH_4_, it compares favorably in terms of equipment cost and portability, and it provides an additional analytical option that enables hydrogen stable isotope-based measurement of methane activation that is complementary to carbon-based stable isotope (^13^C) or radiocarbon (^14^C) methods. As a proof of concept, we apply the monodeuterated-methane approach to pressurized methane seep sediment incubations in order to test the role of an important environmental variable on methanotrophic rates under nontraditional empirical conditions.

## RESULTS AND DISCUSSION

### Comparison of CH_3_D and ^14^CH_4_ rates in aerobic methanotroph cultures.

**FIG 1:**
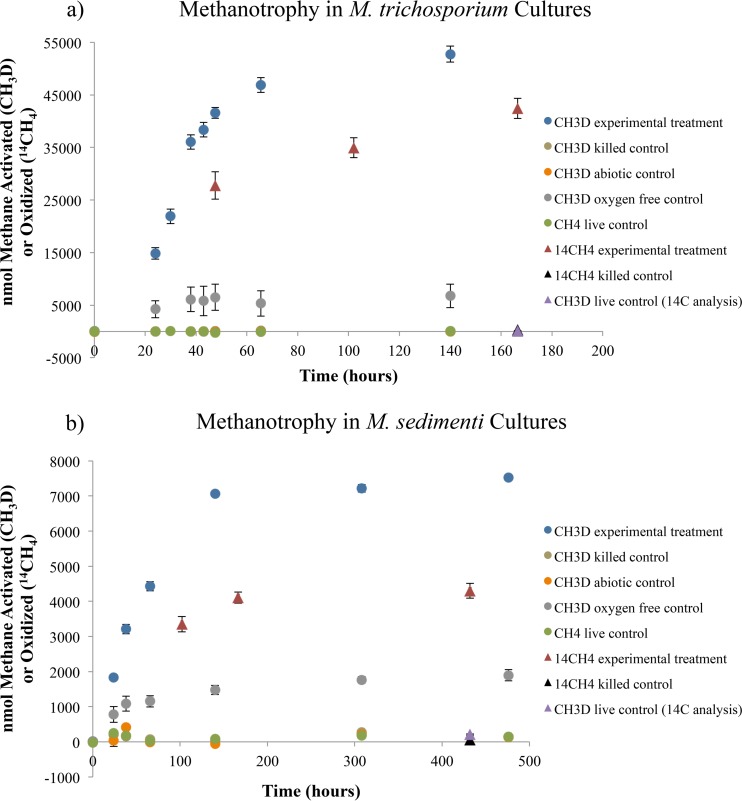
Amount of methane consumed over time for cultures of the type II methanotroph *M. trichosporium* (a) and the type I methanotroph *M. sedimenti* (b) using C_corr_ (values were derived from the CH_3_D method [circles]) and the ^14^CH_4_ method (diamonds), calculated as discussed in the text. ^14^CH_4_-derived data convey values of methane consumption and full oxidation, while CH_3_D-derived data provide a measure of methane activation. Error bars show standard errors for three biological replicates, except for the ^14^CH_4_ killed control (*n* = 1). Obscured data points exhibited values between −60 and 110 nmol for the results in panel a and between 0 and 60 nmol for the results in panel b.

D/H ratios were acquired and corresponding values of methane consumption were calculated at eight points during the Methylosinus trichosporium growth curve and seven points of the Methyloprofundus sedimenti growth curve. Three measurements of ^14^C distributions were acquired for each strain, targeting exponential and stationary phases (Fig. 1). The type II alphaproteobacterial methanotroph *M. trichosporium* exhibited methane consumption rates more than an order of magnitude greater than those of *M. sedimenti* (gammaproteobacterial type I methanotroph), yet the scaling factor relating the CH_3_D- and ^14^CH_4_-derived rates was remarkably consistent in both cases. Scaling factors were calculated for both exponential growth and stationary phase, using data points from both CH_3_D and ^14^CH_4_ experiments. The *M. trichosporium* rate value calculated from the CH_3_D experimental treatment point (47.5 h, 4.16 × 10^4^ nmol of methane consumed) was compared with the rate determined from the ^14^CH_4_ experimental treatment point (47.5 h, 2.78 × 10^4^ nmol of methane consumed), yielding a scaling factor of 1.5 for exponential-phase growth. Similarly, data from the experimental treatment point at 140 h (5.27 × 10^4^ nmol of methane, CH_3_D) and 166.5 h (4.24 × 10^4^ nmol of methane, ^14^CH_4_) were used for *M. trichosporium*’s stationary-phase scaling factor. Equivalent values were determined for *M. sedimenti* using the following data points: 7.07 × 10^3^ nmol of methane after 140 h with CH_3_D and 3.35 × 10^3^ nmol of methane after 102 h with ^14^CH_4_ for the exponential growth phase, and 7.53 × 10^3^ nmol of methane after 476 h with CH_3_D and 4.30 × 10^3^ nmol of methane after 432 h with ^14^CH_4_ for the stationary phase (Fig. 1). It should be noted that simultaneous sampling of CH_3_D and ^14^CH_4_ experiments was not always possible, as they were conducted at different institutions. Nonetheless, the optical density at 600 nm (OD_600_) and rate-based growth curves indicate that all sampling occurred within the designated growth phase (Fig. 1 and see Fig. S1 in the supplemental material).

10.1128/mSphereDirect.00309-17.2 FIG S1 OD_600_ growth curves for cultures of the type II methanotroph *M. trichosporium* (a) and the type I methanotroph *M. sedimenti* (b). Error bars show standard errors for three biological replicates. Download FIG S1, PPTX file, 0.1 MB. Copyright © 2017 Marlow et al. 2017 Marlow et al. This content is distributed under the terms of the Creative Commons Attribution 4.0 International license.

In this way, the ratios of methane consumption rates derived from the CH_3_D method (using equations 1 to 7 [see Materials and Methods]) and the ^14^CH_4_ method (using equation 8) can be compared. This value is herein referred to as the D/^14^C tracer ratio. This ratio can be used to evaluate the consistency of the monodeuterated-methane method compared with the well-established ^14^CH_4_ approach and as a potent investigatory tool to probe the relationship between partial and complete metabolism of methane.

D/^14^C tracer ratio values for aerobic methanotroph cultures tested in this study are shown in Table 1; their consistency is a promising indicator of the utility of the monodeuterated-methane approach. By dividing the methane activation rates derived from D/H values (*R*_CH_3_D_ [see “Rate measurements derived from CH_3_D addition” below]) by 1.5, an estimate of full-oxidation methanotrophy—that is, the complete biological oxidation of methane to carbon dioxide—can be attained.

**TABLE 1:** D/^14^C tracer ratios for the experimental treatments addressed in this study^a^

Sample tested	Ratio in:			
	Exponential phase	Stationary phase	Oxic incubations	Anoxic incubations
Aerobic methanotroph cultures				
*M. trichosporium*	1.5	1.48		
*M. sedimenti*	1.54	1.59		
		
Methane seep sediments and carbonates				
A.Sed-5128			1.62	2.05
L.Sed-5043			1.71	2.01
A.Carb-5305			1.65	1.96
A.Carb-5152			1.63	2.08
L.Carb-5028			1.69	1.86

a Cultures of the aerobic methanotrophs *M. trichosporium* and *M. sedimenti* were tested alongside environmental samples, sediments (Sed) and carbonate rocks (Carb), from Hydrate Ridge methane seeps. “A” refers to sites of active seepage, while “L” indicates locations of low seepage activity, where clear signs of contemporary methane seepage were absent. (See the text for additional sampling details.)

### Comparison of CH_3_D and ^14^CH_4_ rate measurements in environmental methane seep samples.

**FIG 2:**
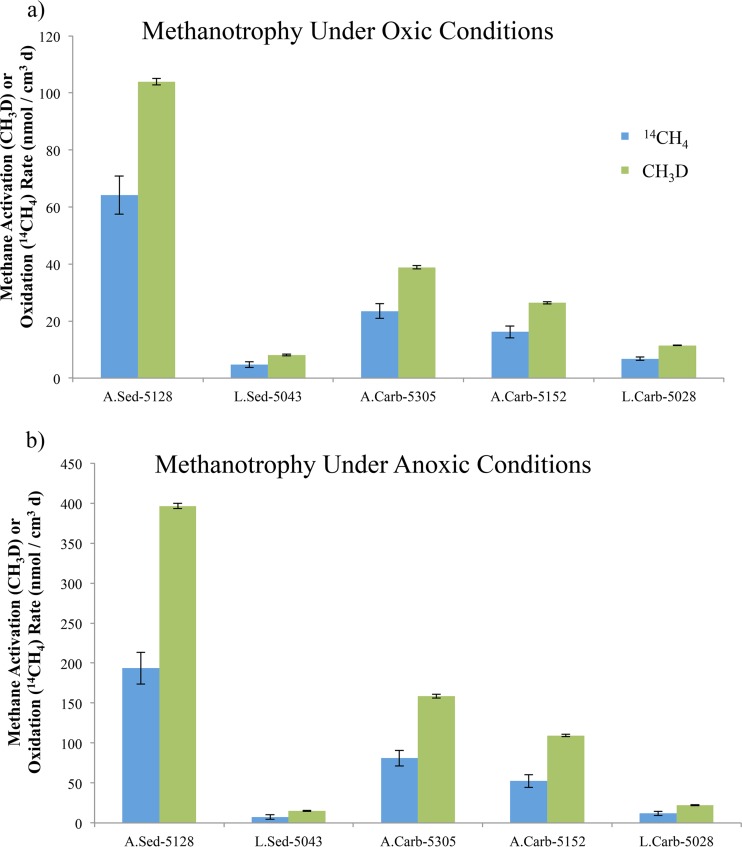
Methanotrophy in oxic (a) and anoxic (b) incubations of active and low-activity seep sediment and carbonate rocks (*n* = 3 in all cases). Values compare rates of methane consumption and full oxidation derived from ^14^CH_4_ measurements (blue) and rates of methane activation derived from the CH_3_D approach (green, *R*_CH_3_D_ values). Values are reflective of rock and initial sediment volumes (not including added water). Rates derived from triplicate A.Sed-5128 killed-control incubations were subtracted from all samples. Standard error bars are provided.

Methane consumption rates under oxic (Fig. 2a) and anoxic (Fig. 2b) microcosm incubation conditions, derived from both CH_3_D and ^14^CH_4_ measurements, are provided for five different sample types from marine methane seeps (active sediment, low-activity sediment, active porous carbonate, active massive carbonate, and low-activity massive carbonate) and were calculated from data collected after 4 days (oxic) or 8 days (anoxic) of incubation.

The D/^14^C tracer ratio was 1.66 ± 0.02 standard error (SE) for the oxic and 1.99 ± 0.04 SE for the anoxic incubations (Table 1). These relatively consistent values across physical substrate type (sediment and carbonates of various lithologies) and collection site activity level (active and low activity) suggest an underlying metabolic basis of the D/^14^C tracer ratio that is unperturbed by physicochemical factors or relative activity levels.

### Understanding the D/^14^C tracer ratio.

The CH_3_D and ^14^CH_4_ approaches quantify distinct aspects of methanotrophy: methane activation and complete conversion to CO_2_, respectively. The ^14^CH_4_ technique quantifies the amount of ^14^C (initially supplied as methane) that is fully oxidized and persists as soluble species (HCO_3_^−^) or acid-labile precipitation products (CaCO_3_). The CH_3_D protocol, on the other hand, reports the extent to which methane-derived hydrogen atoms are detected in water. Abiotic exchange between methane- and water-associated hydrogen atoms is not expected. Indeed, D/H ratios in killed-control experiments remained stable (e.g., exhibiting a value of 1.40 × 10^−4^ ± 3.1 × 10^−8^ SE at time zero [*T*_0_] and 1.40 × 10^−4^ ± 2.9 × 10^−8^ SE at 140 days [*T*_140_] during experimentation with *M. trichosporium* [data are incorporated into Fig. 1a]). The activation of methane thereby indicates enzymatic functionalization, but the ultimate fate of each hydrogen atom during methane oxidation is not known.

**FIG 3:**
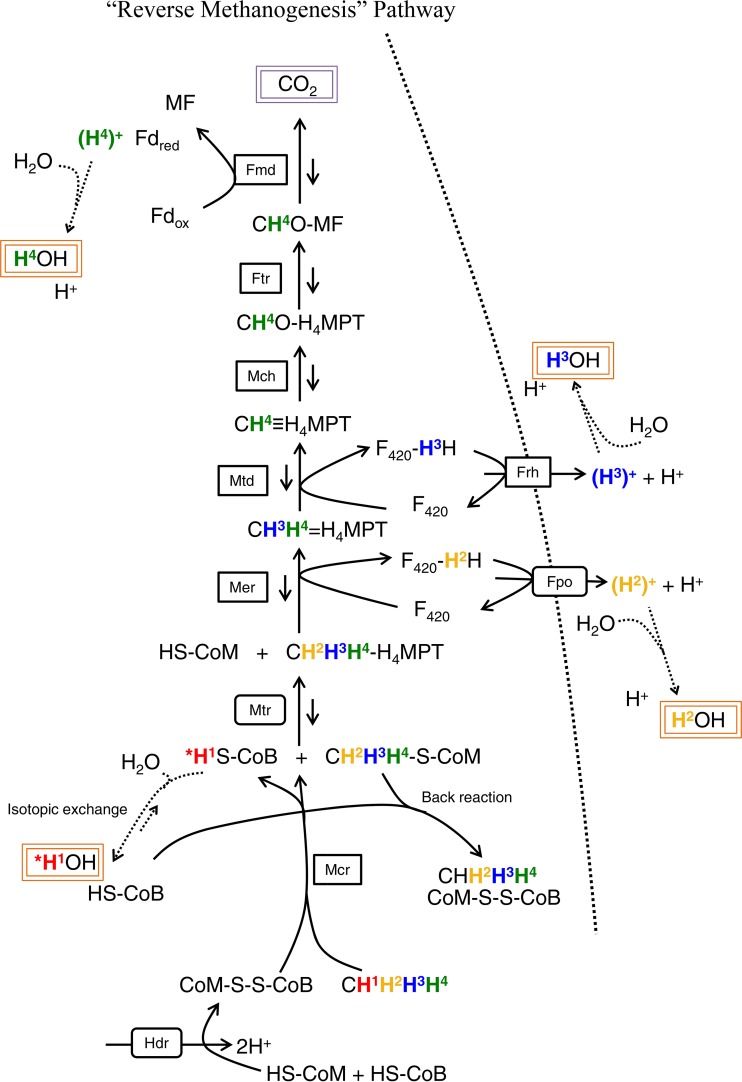
Schematic diagram demonstrating the potential fate of methane-associated hydrogen atoms in the reverse-methanogenesis pathway. Hydrogen atoms are distinguished by color and superscript number, and potential exchanges with inter- and intracellular water are shown; asterisks represent location-specific ambiguity. Potentially detectable methane-derived hydrogen atoms (four, occurring throughout the oxidation pathway) and carbon atoms (one, requiring full oxidation) are highlighted in orange and purple boxes, respectively. Shorter back-flux arrows reflect the observation that all enzymes (85) and the entire pathway (43) have been shown to be reversible. For figure simplicity, not all cofactors or isotopically distinct back-flux products are shown. Enzyme abbreviations are in black-lined boxes, and the extended dashed line represents the cell membrane. Fd_ox_, oxidized ferredoxin; Fd_red_, reduced ferredoxin; MF, methanofuran; H4MPT, tetrahydromethanopterin.

**FIG 4:**
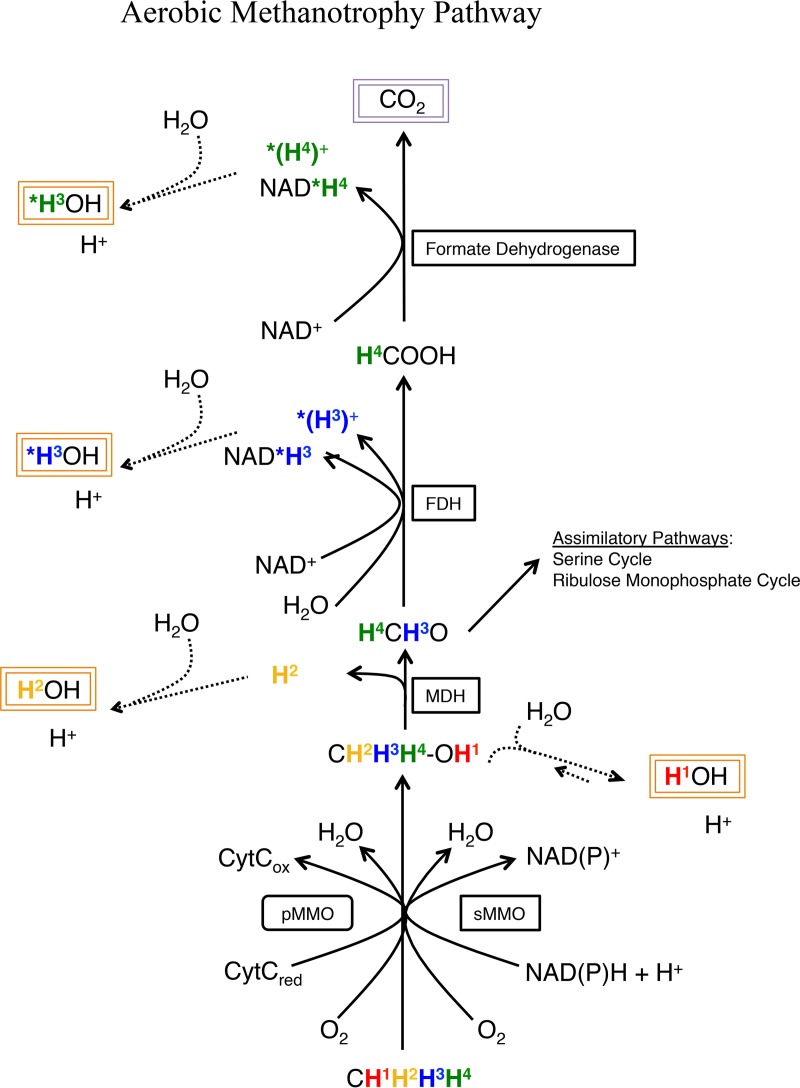
Schematic diagram demonstrating the potential fate of methane-associated hydrogen atoms in the aerobic methanotrophy pathway. Hydrogen atoms are distinguished by color and superscript number; asterisks represent location-specific ambiguity. Potentially detectable methane-derived hydrogen atoms and carbon atoms are highlighted in orange and purple boxes, respectively. Mmo enzymes are not believed to perform reversible reactions. FDH, formate dehydrogenase; CytC_ox_, oxidized cytochrome *c*; CytC_red_, reduced cytochrome *c*; MDH, methanol dehydrogenase; pMMO, particulate methane monooxygenase; sMMO, soluble methane monooxygenase.

The flow of methane-derived hydrogen atoms through anaerobic and aerobic methanotrophic metabolisms was examined in an attempt to predictively evaluate the consequence of monodeuterated-methane reactions. Previously published reports were used to compile Fig. 3 (39–41) and Fig. 4 (42), which trace anaerobic and aerobic methane metabolisms, respectively, with a specific focus on hydrogen atoms. In this context, our observations of relatively consistent but distinct D/^14^C tracer ratios for anaerobic and aerobic methanotrophy (Table 1) likely reflect different aspects of the two metabolic pathways. In AOM, metabolite back-flux (43) may increase the D/H ratio; in aerobic methanotrophy, biomass growth represents a substantial carbon and hydrogen shunt.

### The D/^14^C tracer ratio in anaerobic methanotrophy.

AOM is depicted in Fig. 3 via the reverse-methanogenesis pathway, which is believed to be enacted by anaerobic methanotrophic archaea based on genetic (41, 44, 45) and proteomic (46, 47) data. In this metabolic process, methyl-coenzyme M reductase (Mcr) activates methane and generates methyl-coenzyme M (methyl-CoM). A tetrahydromethanopterin molecule supplants CoM, and subsequent carbon oxidation steps release hydrogen atoms into the medium. Ultimately, the number of methane-derived hydrogen atoms that enter water-exchangeable products determines the physiological interpretation of aqueous D/H ratios. For example, if just one methane-derived hydrogen enters an intermediate and is freely exchangeable with water, then observed water-based deuterium must be multiplied by 4 (to account for methane’s hydrogen-carbon stoichiometry [see equation 5 in Materials and Methods]) and the appropriate primary isotope effect (not evaluated here) to arrive at the actual quantity of activated methane molecules. In this context, the experimental D/^14^C tracer ratio values may provide useful insight. A D/^14^C tracer ratio of 2 for the reverse-methanogenesis pathway suggests that for every methane molecule that is fully oxidized to CO_2_, two hydrogen atoms enter water-exchangeable intermediates.

However, the back-reaction of enzymatic processes (48) may lead to heightened D/H ratios in the absence of full carbon oxidation. For example, upon the activation of methane by Mcr, HS-coenzyme B (HS-CoB) and CH_3_-S-CoM form, with the thiol hydrogen exchanging with water-bound hydrogen. If the initially formed S-bound hydrogen is deuterium, this atom then exchanges with ^1^H from water. Upon Mcr back-reaction, CH_4_ is formed and the aqueous deuterium causes a heightened D/H ratio despite a lack of complete methane oxidation (Fig. 3). We analyzed the remaining headspace of seep sediment incubations for the formation of CH_4_ from CH_3_D via ^1^H-nuclear magnetic resonance (NMR) spectroscopy. Over the course of 58 days in triplicate active-sediment 5128 (A.Sed-5128) incubations prepared with exclusively CH_3_D headspace, CH_4_ in the headspace increased from 0.33% ± 0.02% SE to 4.48% ± 0.27% SE. If this demonstrated reversibility reflects only the back-reaction of Mcr, then the CH_4_ increase (4.15%) must be multiplied by 4 (=16.6%) to reflect the actual percentage of headspace methane that was re-formed by Mcr; if the reversibility reflects back-reaction of the entire pathway, then no scaling factor is needed. Full methane oxidation rates measured via ^14^CH_4_ in different replicates of the same inoculum (Fig. 2b) revealed that 4.1% of the available methane was observed in the fully oxidized state (i.e., as ^14^C-labeled dissolved inorganic carbon [DIC]) during the 58-day incubation and thus did not participate in the back-reaction. An estimated 95.9% of the initial methane remained at the time of NMR measurement, meaning that the amount of initial CH_3_D that may have re-formed as CH_4_ through a partial or complete back-reaction is between 3.98 and 15.92%. For clarity, these calculations neglect isotope effects and activity by methanogens, the latter of which was highly endergonic given the lack of added hydrogen or acetate. These factors can be explored through further experimentation. Reversibility can be evaluated in future stable isotope work by (i) including a [^13^C]DIC source in the water and measuring ^13^CH_4_ and/or (ii) utilizing commercially available multiply deuterated methane as the initial headspace and quantifying all possible isotopologues. Nonetheless, even the upper bound of partially and reversibly oxidized CH_3_D suggests that the majority of the D/H change is attributable to reactions indicative of net methane consumption, if not complete oxidation.

### The D/^14^C tracer ratio in aerobic methanotrophy.

In aerobic methanotrophic cultures, a D/^14^C tracer ratio of ~1.5 was observed, suggesting that on average, 2.67 of the four methane-derived hydrogen atoms likely enter water-exchangeable products during the course of a full-oxidation pathway. *M. trichosporium* is a type II methanotroph, a member of the *Alphaproteobacteria* that uses the serine pathway for carbon assimilation; *M. sedimenti* is a gammaproteobacterial type I methanotroph that uses the RuMP carbon assimilation pathway (49). The pathway data presented in Fig. 4 suggest that all methane-bound hydrogens are water exchangeable during the catabolic oxidation of methane to carbon dioxide. Thus, to achieve a D/^14^C tracer ratio less than 4, a substantial proportion of methane-derived formaldehyde would need to proceed down the assimilatory pathway, a requirement that was likely met given the cultures’ increase in cell density (Fig. S1). Intriguingly, the D/^14^C tracer ratios were similar for the two cultured organisms despite their distinct metabolic pathways; a similar phenomenon of consistent carbon conversion efficiency was recently observed among distinct aerobic methanotroph communities in English riverbeds (50). Previous studies of aerobic methanotrophy compared rates derived from radiolabeled carbon (^14^C)- and hydrogen (^3^H)-based approaches, yielding unpredictable ratios spanning multiple orders of magnitude (29, 51). These findings were attributed to discrepancies in incubation temperatures and metabolic priming effects between methods, highlighting the need for consistent experimental parameters.

The oxic incubations of methane seep sediment produced a D/^14^C tracer ratio of 1.66 ± 0.02 SE. Given that the known modes of biological methane oxidation—type I and type II aerobic methanotrophy and reverse-methanogenesis anaerobic methanotrophy—bound this observed value, it appears likely that the oxic sediment incubations supported a mixture of both aerobic and anaerobic methane oxidation processes. Aerobic methane oxidation likely dominated, based on the ~7 × 10^4^-Pa partial pressure of O_2_ and the proximity of the D/^14^C tracer ratio to that of the aerobic methanotrophic cultures, but anoxic niches likely remained or developed in the incubation bottles.

### Specialized application of monodeuterated methane: examining methane activation under pressure.

To demonstrate the utility of the CH_3_D rate measurement approach in nontraditional empirical contexts, we sought to evaluate the influence of *in situ* pressure on methanotrophic rates of Hydrate Ridge seep sediment microbial communities. Material collected for microbiological studies of AOM is frequently obtained from marine settings of various depths that are subjected to distinct and substantial pressure regimes (52). Pressure is not always rigorously incorporated into microcosm experiments, though evidence suggests that it can be an important determinant of methanotrophic rates (53–56). In addition, some procedural aspects of the ^14^CH_4_ protocol, including headspace sampling and full-volume transfer, are not established for use with Mylar bags, which lack gas-tight sampling ports, making the monodeuterated-methane approach an appealing alternative in this context.

**FIG 5:**
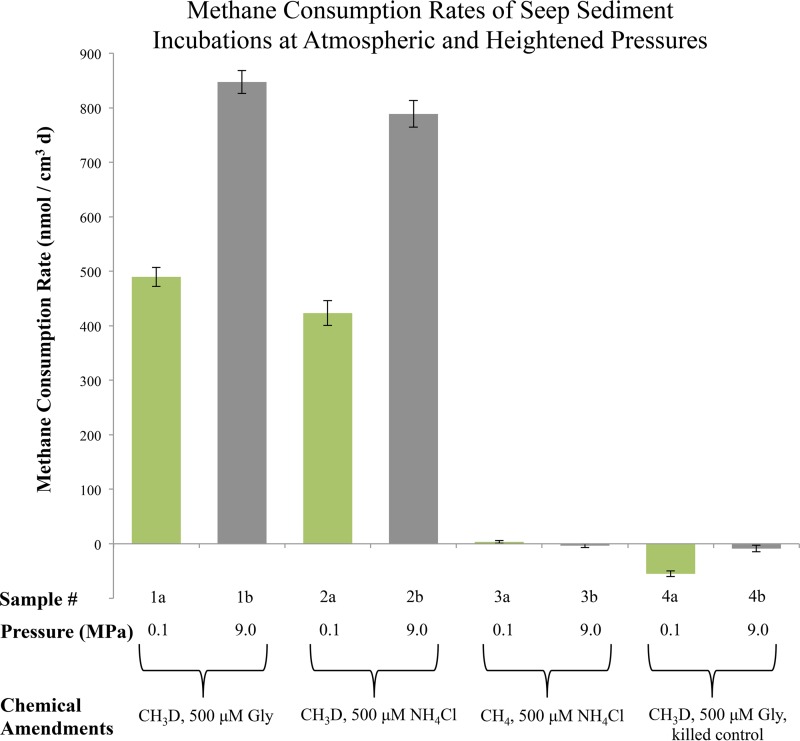
Pressure experiment results showing methane consumption rates derived from aqueous D/H values, with standard error bars, of seep sediment samples following 38-day incubations with CH_3_D at 9.0 MPa (gray bars, “b” samples) or 0.1 MPa (green bars, “a” samples). Additional details on sample treatments can be found in Table S1. Values are reflective of rock and initial sediment volumes (not including added water).

Parallel seep sediment incubations were subjected to 0.1 MPa (atmospheric pressure) and 9.0 MPa (equivalent to an ~900-m depth). Nitrogen in the form of ammonium (500 μM NH_4_Cl) or the amino acid glycine (500 μM) was added to assess whether distinct nitrogen sources influenced AOM rates. Methane consumption rates derived from heightened D/H ratios are shown in Fig. 5. A significant increase in methane consumption was observed under both live conditions at high pressure, corresponding to sediment incubated with glycine (samples 1a and 1b) and ammonium chloride (samples 2a and 2b). Neither live controls lacking CH_3_D (samples 3a and 3b) nor autoclaved, killed controls (samples 4a and 4b) showed activation of CH_3_D (see Table S1 for sample setup details). The simulation of *in situ* Hydrate Ridge pressures led to a 79.5% (±6.5% SE) increase in relative methane consumption rates. Incubation with 500 μM glycine rather than ammonium at high and low pressures resulted in small but consistent rate increases of 12% ± 4.1% SE, potentially reflecting the energetic and biosynthetic distinction between exogenous amino acids and unprocessed fixed nitrogen.

10.1128/mSphereDirect.00309-17.4 TABLE S1 Experimental setup for methane seep sediment pressurized rate measurement incubations. The samples ran for 38 days at 4°C, and each sample was contained in a sealed Mylar bag. Pressure values indicate absolute pressure exerted on the incubated Mylar bags. Download TABLE S1, DOCX file, 0.01 MB. Copyright © 2017 Marlow et al. 2017 Marlow et al. This content is distributed under the terms of the Creative Commons Attribution 4.0 International license.

Previous reports have found a wide range of different pressure-related effects. In a sulfate-coupled AOM bioreactor, pressures were varied from 1 to 8 MPa, and sulfide production approximately tripled (55). Compared with treatment at 0.101 MPa, a 10.1-MPa bioreactor with sediment from Eckernförde Bay demonstrated a cessation of methanogenesis, a 4-fold increase in methane oxidation rates, and high relative abundances of ANME-2a/b and ANME-2c (56). A continuous incubation system with Black Sea microbial mats at 16 MPa measured a 10- to 15-fold increase in methane-dependent sulfide generation compared with ambient pressure (57). Methane partial pressures of 1.1 MPa led to a 5-fold increase in sulfate reduction rates relative to ambient atmospheric pressure with Hydrate Ridge sediments demonstrating methane-dependent sulfate reduction (31). With methane seep sediment from the Japan Trench, however, methane-driven sulfate reduction rates did not correlate with changing pressure (58). Nauhaus et al. (54) suggested that the pressure-induced rate increases are due more to heightened methane solubility and bioavailability than to physiological effects or biomolecular reordering. Bowles et al. (53) presented a very different perspective by showing a 6- to 10-fold AOM rate increase at 10 MPa when methane concentrations were held constant. Deconvolving these two influences and how they depend on community composition or physicochemical parameters is feasible with pressure chamber experiments utilizing monodeuterated methane. Intriguingly, in combination with a carbon-based isotopic probe, aqueous D/H measurements might be used to evaluate predictions that AOM at heightened pressure exhibits decreased back-fluxes (39, 59) and, if so, whether the barrier occurs after complete or partial oxidation. More broadly, understanding the relative contributions of environmental and physiological effects to methane oxidation will help constrain methane fluxes across a large envelope of the planet’s methanotrophically active zones.

### Using monodeuterated methane in experimental investigations.

Based on ^14^CH_4_ ground truth experiments with aerobic methanotrophic cultures, oxic seep sediment, and anoxic seep sediment, as well as the proof-of-concept pressurized experiments, we believe that the monodeuterated-methane approach to methane oxidation rate measurement is a useful addition to a biogeochemist’s tool set (Table 2). Compared with radiolabel approaches (^14^CH_4_, [^3^H]CH_4_, ^35^SO_4_^2−^), the method requires less safety-oriented planning and is procedurally simpler, more affordable, and less susceptible to hydrogen-associated isotope fractionation effects (relative to ^3^H). Our results also suggest that the monodeuterated-methane technique appears to be a more precise method based on standard error calculations (Fig. 1 and 2; Table S2). Direct comparisons of environmental incubations are complicated by the microheterogeneity of seep settings (60, 61), as well as the fact that different aliquots of the same initial material were used in our experiments. Analysis of culture-based and seep substrate experiments reveals that standard errors from CH_3_D-derived values were between 1.56 times lower (*M. trichosporium* cultures) and 4.76 times lower (anoxic seep substrate incubations) than those derived from ^14^CH_4_-based values (Table S2).

10.1128/mSphereDirect.00309-17.5 TABLE S2 Standard errors from experiments comparing CH_3_D- and ^14^CH_4_-derived measurements. For aerobic methanotroph cultures (data shown in Fig. 1), experimental treatments were compared, and ratios of standard errors were compared between the closest available time points (designated by color shading). For seep sediment substrates (data shown in Fig. 2), endpoint rates were used for comparison; the SE for CH_3_D/SE for ^14^CH_4_ ratios do not change if overall quantities rather than rates are used. Mean ratios of the standard errors derived from the two methods are provided as well for the different experiments. The reciprocal of these ratios indicates how much more precise the numerator method is than the denominator method. For example, for anoxic seep substrate incubations, the CH_3_D approach is 4.76 times more precise (1/0.21) than the ^14^CH_4_ method. Download TABLE S2, DOCX file, 0.1 MB. Copyright © 2017 Marlow et al. 2017 Marlow et al. This content is distributed under the terms of the Creative Commons Attribution 4.0 International license.

**TABLE 2:** Brief summary of the features and potential challenges associated with some of the most prominent methods of experimental methane rate assessment

Method	Feature(s)	Challenges
Methane concn measurements	Directly measures net methane consumption or production	Low sensitivity; limited information on metabolic end product

[^14^C]CH_4_	High sensitivity; tracks carbon atoms and can quantify full methane oxidation; applicable to intact sediment cores; allows high-throughput sampling in the field	Radiolabel faces health and safety regulations; processing samples is procedurally time-intensive

^13^CH_4_	Tracks carbon atoms and can quantify anabolic and catabolic processes, including full methane oxidation	Naturally occurring dissolved inorganic carbon pools can complicate experiments; not yet tested for intact sediment cores

[^3^H]CH_4_	High sensitivity; high specific activity; tracks hydrogen atoms; allows high-throughput sampling in the field	Radiolabel faces health and safety regulations; not practicable for sediment systems; inconsistent relationship with carbon-based rate measurements

CH_3_D	Measures methane activation; tracks hydrogen atoms to enable a better understanding of methane metabolism; logistically and procedurally straightforward; high measurement precision	Hydrogen atom dynamics in methane metabolisms are not fully known; not yet tested for intact sediment cores

Because the monodeuterated-methane method focuses on methane-bound hydrogen atoms, it offers information about methanotrophic systems that is different from and yet complementary to that offered by carbon-based techniques like ^13^C stable-isotope tracking or quantification of methane or bicarbonate. While this distinction complicates the interpretation of isolated D/H ratios, it can offer additional information for analysis of methane-derived intermediates in relevant metabolisms. Given these caveats, we recommend three applications for monodeuterated methane in methane oxidation rate measurement studies.

First, the approach can be employed in a strictly comparative context using an analogous inoculum exposed to a range of different conditions, as demonstrated with the pressure-based sediment incubations presented above. Promising applications include evaluating the effect of different conditions such as temperature ranges, chemical concentrations, or energetic landscapes on seep sediment methane-oxidizing rates. Comparative analysis of rates at different seep sites would also be useful, provided anaerobic or aerobic methanotrophic processes could be isolated.

Second, by performing side-by-side monodeuterated-methane and radiocarbon tests, a sample-specific D/^14^C tracer ratio can be determined, and estimated rates of complete methane oxidation can then be assessed in subsequent experiments on aliquots of the same initial sample material using CH_3_D. Conducting such paired studies under additional environmental or lab-based conditions would help clarify the universality of the ratios presented here. In particular, maintaining consistent headspace proportions and ensuring full equilibration between phases in cultures and incubations would eliminate two potential sources of uncertainty. Mohr et al. (62) showed that more than an hour of continuous shaking was needed to approach full equilibrium dissolution of N_2_ gas and that nitrogen fixation rates had traditionally been underestimated as a result. If similar kinetics govern methane solubility, shorter incubations might have artificially low D/^14^C tracer ratios, though such patterns were not observed between exponential and stationary phases of aerobic methanotroph culture experiments (Table 1). Although initial dissolved methane concentrations were equivalent between the CH_3_D and ^14^CH_4_ experiments, the larger overall quantity of methane available to CH_3_D incubations with headspace may have enabled a more exergonic methane-oxidizing metabolism as the experiments progressed. Further interrogation of these variables would help to clarify their relative importance while providing a robust framework for application of the CH_3_D technique to each user’s experimental system. In addition, experiments with the intra-aerobic pathway of “Candidatus Methylomirabilis oxyfera” (63, 64) or nitrate- or metal-reducing methanotrophic metabolisms (18, 20, 21) would be valuable contributions, as would the extension of the approach to other experimental setups, such as intact sediment cores. We also encourage side-by-side comparisons with other rate measurement approaches, including [^3^H]CH_4_ radiotracer and methane concentration assessments, to develop additional pairwise conversion factors and better constrain carbon and hydrogen metabolism in methane-based biological reactions.

Finally, the use of monodeuterated methane as an analytical tool, alongside additional methods, such as carbon- or sulfur-tracking procedures, would enable the examination of anabolic and catabolic processes in methane-based metabolisms using multiple types of atoms. In particular, the D/^14^C tracer ratios presented here reveal intriguing and seemingly systematic relationships between carbon and hydrogen anabolic and catabolic partitioning across distinct physiologies, yet an underlying theoretical framework regarding the fate of methane-bound hydrogen atoms remains outstanding. In anaerobic methanotrophic systems, back-reaction rates and equilibrium constants might be evaluated by (i) including a ^13^CO_2_ source in the water and measuring ^13^CH_4_, (ii) using ^13^CH_4_ in the headspace and quantifying its dilution by ^12^CH_4_ produced during back-reaction (28, 65), or (iii) adding multiply deuterated methane as the initial headspace and measuring all possible isotopologues via NMR or high-resolution mass spectrometry. Tracking sulfur and oxygen isotopic distributions of sulfate can characterize the back-flux of sulfate reduction (59, 66); linking this process with methane oxidation and back-reaction would provide insight into the close metabolic coupling between ANME and SRB. For aerobic methanotrophs, evaluating D/^14^C tracer ratios under more clearly defined growth and maintenance phases would elucidate distinct values associated with catabolic, RuMP, and serine pathways, enabling future use of that parameter as an arbiter of relative anabolic and catabolic activities. Furthermore, additional environmental variables can be tested to gain insight into distinct redox pathways and dynamics of reversibility. For example, under low sulfate concentrations, back-flux of the AOM reaction increases as methane and carbon dioxide approach carbon isotopic equilibrium (67), and a higher D/^14^C tracer ratio might be expected. In this context, the D/^14^C tracer ratio could be further developed as a measure of microbially mediated isotopic equilibration.

### Conclusions.

The ability to accurately measure methane consumption and oxidation rates—both comparatively and in absolute values—is an important component of methanotrophic studies. Such measurements frequently depend on radiotracers or measurements of chemical species that are related to, but not directly indicative of, methane metabolism. The monodeuterated-methane technique presented here represents a novel approach to investigate methane oxidation rates, notable for its logistical ease and straightforward sampling procedures. We have demonstrated that the D/H ratio is a reliable proxy for methane oxidation activity when subjected to ground truth experiments on a sample-specific basis with the well-established ^14^CH_4_ method; in several applications, methane consumption values calculated via the CH_3_D method were directly proportional to ^14^C radiolabel-derived methane oxidation rates. Values of the proportionality constant differ based on the experimental system, likely dictated by environmental variables and the relative proportions of aerobic and anaerobic methanotrophic metabolisms, though additional experiments to determine the nature of the putative mixing line are needed. By providing a way to measure how hydrogen atoms are mobilized and processed, deuterated methane represents a promising approach to help researchers disentangle several aspects of methane-associated metabolisms.

Methane biogeochemistry is a dynamic field of study with implications for carbon cycling, microbial ecology, and climate dynamics, though experimental challenges have slowed our understanding of methane-based biological reactions. With the CH_3_D approach as an added tool in the arsenal of rate-based examinations, a broader understanding of the intricacies of methane metabolism, as well as its role in environmental and anthropogenic systems, is within reach.

## MATERIALS AND METHODS

### Experimental setup.

To demonstrate the precision and reproducibility of the monodeuterated-methane approach, it was tested alongside the well-established ^14^CH_4_ radiotracer protocol. The use of ^14^CH_4_ is an accepted standard procedure in studies of methane consumption quantification (68–71) and has been experimentally cross-referenced with methane concentration measurements (37) and other approaches, including tritiated-methane techniques (29, 51). Both techniques were applied to (i) aerobic methanotrophic cultures of Methylosinus trichosporium OB3b (kindly supplied by Marina Kalyuzhnaya and Mary Lidstrom) and Methyloprofundus sedimenti (isolated from a deep sea whale fall [49]); (ii) oxic incubations of methane seep sediment slurries and carbonate rocks, and (iii) anoxic incubations of methane seep sediment slurries and carbonate rocks. In addition, the monodeuterated-methane protocol was employed in a pressure-based experiment to demonstrate the technique’s adaptability to distinct empirical setups and to examine the relative effects of high, environmentally relevant pressures on methane consumption rates in anoxic seep sediment samples. Monodeuterated-methane gas for all samples was 98% pure CH_3_D (the remainder was CH_4_, air, CO_2_, and C_2_H_6_) obtained from Sigma-Aldrich ($247/liter). For a representation of all experiments conducted in this study, see Table 3.

**TABLE 3:** Summary of the samples used for all experiments conducted in this study^a^

Expt and sample	Experimentation under oxic conditions		Experimentation under anoxic conditions	
	CH_3_D	^14^CH_4_	CH_3_D	^14^CH_4_
Aerobic methanotroph cultures				
*M. trichosporium*	×	×		
*M. sedimenti*	×	×		

Seep sediments				
A.Sed-5128	×	×	×	×
L.Sed-5043	×	×	×	×

Seep carbonates				
A.Carb-5305	×	×	×	×
A.Carb-5152	×	×	×	×
L.Carb-5028	×	×	×	×

Seep sediment at pressure				
A.Sed-3450			×	

a Cells with exes indicate that the experiment took place (with all relevant permutations and controls, as described in the text); blank cells indicate experiments that were not conducted. CH_3_D refers to the methanotrophic rate in experiments using the novel monodeuterated-methane technique, while ^14^CH_4_ refers to the radiolabel-based experiments. The three-part codes for samples derived from environmental material refer to active (A) or low-activity (L) sediments (Sed) or carbonates (Carb), along with a sample-specific four-digit serial number.

### Experiments with aerobic methanotroph cultures.

Cultures of Methylosinus trichosporium strain OB3b were grown using nitrate mineral salts (NMS) medium at 30°C (72). The newly characterized Methyloprofundus sedimenti strain WF1 was grown in a modified NMS medium at 25°C (49). In both cases, shaking cultures were grown up from stock in sealed 25-ml test tubes that contained 5 ml medium and 50:50 air:methane by volume. After several successful transfers (as determined by an increase in optical density [data not shown]), experiments were initiated by passaging 0.94 ml of exponential-phase inoculum into 25-ml glass Balch tubes containing 8.5 ml medium, resulting in a final volume of 9.44 ml. The headspace was adjusted to result in the dissolved methane, oxygen, and argon concentrations shown in Table S3 in the supplemental material for each of 10 different experimental conditions. CH_3_D experiments were run with headspace. ^14^CH_4_ experiments were performed without headspace: medium was preinoculated and preequilibrated in 25-ml tubes with relevant gases (with the exception of the dissolved ^14^CH_4_ tracer) to ensure that initial reactant concentrations were consistent between different rate measurement techniques. This medium was then transferred by gas-tight syringe to separate 10-ml test tubes (measured to hold 9.44 ml when stoppered, allowing for volumes equivalent to those used in the CH_3_D experiments) while simultaneously removing headspace until no headspace was present. All resulting treatments were prepared in triplicate, and all tubes were sealed by blue rubber chlorobutyl stoppers (Bellco Glass, Inc., Vineland, NJ) and aluminum crimp caps. Due to the destructive nature of the ^14^CH_4_ method, methane oxidation measurements at each of three distinct time points required dedicated triplicate sets of culture (Table S3).

10.1128/mSphereDirect.00309-17.6 TABLE S3 Conditions for the aerobic methanotrophy experiments. All sample types were set up in triplicate (with the exception of sample 10); samples 1 to 5 were used for aqueous D/H analysis, and samples 6 to 10 were used for ^14^C analysis. Dissolved concentrations were calculated based on Henry’s law constants compiled by the National Institute of Standards and Technology and adjusted based on the temperature and salinity conditions (1) of growth conditions (2, 3). Millimolar concentrations for *M. trichosporium* (*Mt*) and *M. sedimenti* (*Ms*) experiments are provided. Download TABLE S3, DOCX file, 0.01 MB. Copyright © 2017 Marlow et al. 2017 Marlow et al. This content is distributed under the terms of the Creative Commons Attribution 4.0 International license.

One-milliliter subsamples of fluid for D/H analysis were taken (by syringe through the stopper) at seven time points throughout the 140-h (*M. trichosporium*) and 476-h (*M. sedimenti*) experiments. Samples for radiolabel processing (full protocol details are provided below) were taken at 47.5, 102, and 166.5 h for *M. trichosporium* cultures and 102, 166.5, and 432 h for the slower-growing *M. sedimenti* cultures. Autoclave-killed, cell-free, oxygen-free, and label-free controls were all assessed (Table S3). Sampling points were concentrated around anticipated exponential growth phases as determined by optical density profiles of earlier rounds of culture transfers (measured by determining optical density at 600 nm [OD_600_] using a Beckman Coulter DU 800 spectrophotometer). During the aerobic methanotrophy rate experiments, OD_600_ was measured to confirm culture growth (Fig. S1).

### Experiments with environmental samples: methane seep sediment slurries and carbonates.

Samples recovered from the Hydrate Ridge methane seep system were used to comparatively examine the novel monodeuterated-methane (CH_3_D) approach alongside the ^14^CH_4_ protocol with environmental samples. Hydrate Ridge, OR, is located along a convergent tectonic margin and is well established as a site of methane seepage and sediment-based AOM (37, 73–75). Methane concentrations within the most active seep sediments reach concentrations of several millimolar and have been measured and modeled at values up to 70 mM (76) and 50 mM (74), respectively.

Samples were collected with the deep-submergence vehicle (DSV) *Alvin* during *Atlantis* leg AT-16-68 in September 2010 and the remotely operated vehicle (ROV) *Jason II* during *Atlantis* leg AT-18-10 in September 2011; materials used for methanotrophic-rate experiments are specified in Table 3. The “active” designation in our sample descriptions refers to sites where methane seepage was manifested by seafloor ecosystems known to be fueled by subsurface methane (e.g., clam beds and microbial mats) or methane ebullition (37, 77). The term “low activity” refers to sampling sites that did not exhibit any clear signs of contemporary methane seepage or chemosynthetic communities, though a small amount of methane supply and methanotrophic potential cannot be ruled out, as subsurface advective flow can shift with time (74, 75, 78). Samples spanned a range of physical substrate type (sediment versus carbonate rock) and seepage environments (active and low activity), and are abbreviated by the A.Sed (active sediment), A.Carb (active carbonate), L.Sed (low-activity sediment), and L.Carb (low-activity carbonate) designations. Carbonate samples include both porous materials with macroscale vugs and pore spaces, as well as massive lithologies with a more homogenous structure.

Shipboard, push cores, and bottom water-submerged carbonates were immediately transferred to a 4°C walk-in cold room and processed within several hours. Sediment and carbonate rocks were stored in anoxic, Ar-flushed, gas-tight Mylar bags (IMPAK Corp., Los Angeles, USA) at 4°C until use several months later. In advance of the experimental setup, carbonate samples and homogenized sediment from the 0- to 12-cm push core horizon were prepared under anoxic conditions using 0.22-μm-filtered, anoxic N_2_-sparged Hydrate Ridge bottom water (at a 1:2 sediment/carbonate to bottom water ratio by volume). Samples were maintained under a 2 × 10^5^ Pa CH_4_ headspace for 1 month to resuscitate activity; the corresponding dissolved concentration (1.1 mM, calculated using a temperature-adjusted Henry’s law constant of 5.7 × 10^−6^ [79]) is consistent with the lower range (1 to >50 mM) of methane concentration measured at chemosynthetically active sites at Hydrate Ridge (80).

For the experimental incubations, 10 ml of physical substrate (consolidated sediment or carbonate rock) and 20 ml of filtered Hydrate Ridge bottom water were placed in 60-ml glass bottles (SVG-50 gaschro vials; Nichiden Rika Glass Co., Kobe, Japan). Rate measurements were calculated using the volume of initial consolidated sediment or carbonate rock. In all experiments involving carbonates, interior portions (>5 cm from the rock exterior) were used in order to ensure that properties exhibited were representative of bulk carbonate material and not a reflection of surface-based adherent cells. These interior subsamples were collected using an ethanol-sterilized hammer and chisel. Subsequently, subsamples were fragmented in order to fit through the 28-mm-diameter bottle opening; pieces were kept as large as possible to minimize the increase in surface area-to-volume ratio and maintain conditions as representative of the *in situ* environment as possible. All bottles were sealed with rubber butyl stoppers and twist-on plastic caps.

For CH_3_D experiments, incubations were sparged for several minutes each with N_2_ gas and then methane (CH_4_) gas. Next, an additional 30 ml of gas, whose composition varied depending on the experiment, was injected into the 30-ml headspace to generate an absolute pressure of approximately 2 × 10^5^ Pa. The anoxic-incubation headspace was 2 × 10^5^ Pa methane (50:50 CH_3_D:CH_4_); the oxic-incubation headspace was 1 × 10^5^ Pa methane (50:50 CH_3_D:CH_4_), 6.7 × 10^4^ Pa N_2_, and 3.3 × 10^4^ Pa O_2_. For the radiolabel experiments, 0.22-μm-filtered, anoxic N_2_-sparged Hydrate Ridge bottom water was preequilibrated with similar headspace compositions: 2 × 10^5^ Pa methane (CH_4_) for the anoxic treatments and 1 × 10^5^ Pa methane (CH_4_), 6.7 × 10^4^ Pa N_2_, and 3.3 × 10^4^ Pa O_2_ for the oxic treatments. This medium was then injected via gas-tight syringe into the radiolabel experiment bottles (Ar-sparged bottles with inoculum material) to maintain initial concentrations equivalent to those of the CH_3_D experiments and headspace-free conditions. ^14^CH_4_ was added in quantities detailed in “Rate measurements derived from ^14^CH_4_ addition” below.

All incubation setup prior to gas flushing, headspace injection, and medium transfer took place in an anaerobic chamber. Triplicate samples, including autoclaved killed controls, were prepared for all sample types. Measurements were taken for both D/H and ^14^C analysis at 46 and 96 h for oxic incubations and at 72 and 192 h for anoxic incubations, respectively. Anoxic active methane seep sediment (A.Sed-5128) incubations were used for nuclear magnetic resonance (NMR) analysis of the remaining methane as well as studies assessing the resolution of the CH_3_D method (Text S1 and Fig. S2).

10.1128/mSphereDirect.00309-17.1 TEXT S1 Resolution of the monodeuterated-methane measurement approach and effect of storage on D/H values of sampled water. Download TEXT S1, DOCX file, 0.02 MB. Copyright © 2017 Marlow et al. 2017 Marlow et al. This content is distributed under the terms of the Creative Commons Attribution 4.0 International license.

10.1128/mSphereDirect.00309-17.3 FIG S2 To assess the empirical resolving power of the D/H measurement technique, we determined the time points showing nonoverlapping confidence intervals for triplicate incubations of A.Sed-5128. Each replicate is shown in a different panel, with horizontal extensions of the first time point’s confidence interval. Statistically distinct signals were seen at the 20-h time point for replicates A and B (green and teal diamonds) and the 26-h time point for replicate C (pink diamonds). Download FIG S2, PPTX file, 0.1 MB. Copyright © 2017 Marlow et al. 2017 Marlow et al. This content is distributed under the terms of the Creative Commons Attribution 4.0 International license.

### Experiments with environmental samples in pressure vessels.

The monodeuterated-methane technique was used to determine the effect of pressure on anaerobic methanotrophic rates. Active sediment from Hydrate Ridge (A.Sed-3450) was collected from a water depth of 850 m and an ambient temperature of 4°C, processed shipboard, and prepared for experimentation as described in “Experiments with environmental samples: methane seep sediment slurries and carbonates” above.

To set up the incubations, eight Mylar bags were filled with 50 ml homogenized sediment slurry from the 0- to 12-cm horizon (prepared at a 1:2 ratio of consolidated sediment to anoxic bottom water, by volume) and 40 ml methane (Table S1). Glycine (500 μM) or ammonium (500 μM) was added in order to evaluate relative rate differences associated with organic and inorganic sources of nitrogen. Duplicate sets of each of the four sample types, including autoclaved killed controls, were subjected to low pressure (0.1 MPa, i.e., atmospheric pressure) and high pressure (9.0 MPa, equivalent to an ~900-m water depth) (Table S1). Prior to gas addition, each bag was flushed for 5 min with Ar.

The use of flexible Mylar bags is essential for the application of external pressure, yet it presents obstacles for “traditional” methanotrophic rate measurement protocols, such as the ^14^CH_4_ method. In particular, the processing of postincubation headspace is optimized for stoppered bottles, and accessing the gas phase from Mylar bags in a quantitative fashion is challenging. Measurement of radiolabeled dissolved inorganic carbon requires that all incubation material be transferred to an Erlenmeyer flask equipped with a scintillation vial; sediment grains are commonly trapped in the seals of Mylar bags, complicating this transfer. For these reasons, monodeuterated-methane addition and subsequent aqueous measurement offered a useful tool for this challenging experimental setup.

The incubation mixtures were prepared and placed in a walk-in cold room (4°C). Incubations for pressurized treatment were inserted into a stainless steel, custom-built pressure chamber with 3-cm-thick walls, and hydraulic fluid was pumped into the sealed chamber using a Star Hydraulics P1A-250 hand pump. The pressure was maintained at 9.0 MPa during the course of the 38-day experiment, with daily adjustments to account for thermal compression effects. At the conclusion of the experiment, Mylar bags were removed from the chamber and checked for leaks by evaluating positive pressure inflation and determining if any hydraulic fluid had entered the bags. Upon confirmation that no leaks had occurred, the bags were sampled for D/H ratio measurement.

### Analytical procedures. (i) Rate measurements derived from CH_3_D addition.

At designated sampling times, 1 ml of medium or seawater was collected from cultures or sediment/carbonate samples, respectively, in an anaerobic chamber with a sterile syringe through a gas-tight stopper. A constant volume was maintained by adding 1 ml of sterile medium immediately after sampling. This medium was preequilibrated with a gaseous headspace specific to each experiment, reflecting the N_2_, CH_4_, CH_3_D, and/or O_2_ partial pressures of the corresponding treatment. Medium was not supplemented in the pressure experiment incubations. Sampled liquid was pushed through a 0.22-μm Durapore filter (EMD Millipore, Temecula, CA) and into a 1-ml gas chromatography (GC) vial. A DLT-100 liquid water isotope analyzer (LWIA) (Los Gatos Research, Mountain View, CA) was used to determine the D/H ratio of each sample. The LWIA uses off-axis integrated-cavity output spectroscopy to measure isotopically specific absorption patterns and determine simultaneous D/H and ^18^O/^16^O ratios with high precision (81). Such instruments have been used for a range of studies, including hydrological analysis (82), mine waste management (83), and microbial metabolism (84). We assessed the potential influence of long-term storage on D/H values and determined that values changed by less than 0.5% over 132 days when stored in GC vials at 4°C (Text S1 and Table S4).

10.1128/mSphereDirect.00309-17.7 TABLE S4 D/H ratios and the corresponding calculated quantity of activated methane of water samples stored at 4°C for 132 days. Samples from the *M. trichosporium* aerobic methane oxidation experiment, initially sampled at the 65.5-h time point, were used; this corresponds to the 0-day time point in this table. Individual tubes, rather than triplicates, were used to most clearly determine whether drift in D/H ratios occurred due to storage. Download TABLE S4, DOCX file, 0.1 MB. Copyright © 2017 Marlow et al. 2017 Marlow et al. This content is distributed under the terms of the Creative Commons Attribution 4.0 International license.

In this study, an injection volume of 700 nl at 1,000 nl/s was used, with four intrainjection flush strokes and a flush time of 60 s between injections. Four rounds of 10 injections per sample were performed; to avoid memory effects, i.e., the retention or carry-over of the previous analyte, only the last five injections from each round were used in subsequent calculations. Each analysis included an appropriate blank: (i) autoclaved medium for the cultures or (ii) filter-sterilized bottom water used during the incubation setup of sediments and carbonates. Additionally, two standards of known isotopic ratios were included (deep blue, δD = 0.5‰, and California Institute of Technology standard, δD = −73.4‰). Data were excluded, and D/H ratios were calculated using the remaining measurements, if instrumental temperature or pressure parameters were observed to fall outside optimal instrument specifications (0.76% of all analyses), corresponding to an internal temperature change of more than 0.3°C per h or rising pressure within the measurement cell during the analysis. On average, 12 samples were analyzed during each 7-h run, involving minimal preparation time (~20 min) before loading of samples on the instrument. The procedure described here represents a conservative approach for assessing instrumental drift and statistical validity, and it is likely that the process can be further streamlined.

To calculate methane consumption rates, D/H ratios were first normalized to the Vienna standard mean ocean water (VSMOW) scale using a two-point calibration from the water standards and a linear interpolation (84). To minimize the effects of instrumental drift, standards were remeasured after every 40 injections and new scaling factors were implemented. The number of total moles of hydrogen (H and D) present at the start of the experiment (*T*_1_) prior to CH_3_D addition was calculated using the experiment’s overall water volume, as in equation 1.

(1) $$\frac{\text{volume}\;\left(\text{liter}\right)}{1}\times \frac{\text{55.5 mol water}}{\text{liter}}\times \frac{\text{2 mol hydrogen}}{\text{moles of water}}=\text{moles of hydrogen in incubation at }T_{1}\;$$

The number of D moles newly present in the experiment’s aqueous phase (D_new_) between time points *T*_1_ and *T*_2_ (time 2) was determined using the normalized D/H values (equations 2 to 4).

(2) $$\left[{\left(\frac{\text{D}}{\text{H}}\right)}_{T_{2}}\times {\text{H}}_{T_{2}}\text{(moles)}\right]-\left[{\left(\frac{\text{D}}{\text{H}}\right)}_{T_{1}}\times {\text{H}}_{T_{1}}\left(\text{moles}\right)\right]=\text{new D in incubation }\left(\text{moles}\right)={\text{D}}_{\text{new}}$$

(3) $${\text{H}}_{T_{2}}\approx {\text{H}}_{T_{1}}\approx \text{moles of hydrogen in incubation at }T_{1}$$

(4) $$\left[{\left(\frac{\text{D}}{\text{H}}\right)}_{T_{2}}-\;{\left(\frac{\text{D}}{\text{H}}\right)}_{T_{1}}\right]\times {\left(\text{moles of hydrogen in incubation}\right)}_{T_{1}}={\text{D}}_{\text{new}}$$

D_new_ was multiplied by 4 given the 1:3 D-to-H stoichiometry of the CH_3_D substrate to derive the maximum number of methane molecules consumed catabolically through initial C–X bond activation (equation 5).

(5) $${\text{D}}_{\text{new}}\times 4=\text{maximum moles of methane consumed}=C$$

The scaling factor of 4 was used in the context of methane activation (the initial mobilization through conversion to a methyl group) to calculate the maximum number of methane molecules that could be consumed but not necessarily fully oxidized. This represents an end-member case that may not be appropriate for all metabolic scenarios, as hydrogen/deuterium atoms are exchanged or taken up into biomass. Fractionation factors were not incorporated into the calculations above, as their values are not well constrained for all methanotrophic pathway reactions. Caveats and potential interpretations of the absolute numbers that result from these calculations are discussed above, but we stress that with consistent implementation of scaling factors from sample- or site-specific comparisons between monodeuterated and radiolabel methods, rates derived from *C* are valid and useful.

*C* was corrected based on the fraction of incubation methane headspace composed of CH_3_D, yielding *C*_corr_, as shown in equation 6.

(6) $$\frac{C}{{\text{fraction of methane headspace that is CH}}_{3}\text{D}}=C_{\text{corr}}$$

By dividing *C*_corr_ by the incubation time and sample volume (e.g., liquid culture, consolidated sediment, rock fragment), a maximum rate of methane consumption is determined. The value is converted to nanomoles per cubic centimeter per day, the units most commonly reported in methane metabolism rate studies (equation 7).

(7) $$C_{\text{corr}}\times \frac{{\text{10}}^{\text{9}}\text{ nmol}}{\text{mol}}\times \frac{1}{\text{incubation time (days)}}\times \frac{1}{{\text{incubation volume (cm}}^{3}\text{)}}=R_{{\text{CH}}_{3}\text{D}}$$

where *R*_CH_3_D_ is the maximum rate of methane consumption in nanomoles per cubic centimeter per day.

### (ii) Rate measurements derived from ^14^CH_4_ addition.

Methane oxidation rates using a radiolabeled methane substrate were measured as described in detail by Treude et al. (71). Headspace-free incubations were set up as described above, and radiolabeled methane was injected into each sample container (^14^CH_4_ dissolved in seawater, with a specific activity of 2.07 GBq/mmol and an activity of 13 kBq for culture experiments and 52 kBq in sediment and carbonate samples). To stop microbial activity and begin analysis, 2.5 ml of 2.5% NaOH was injected. Sample headspace flowed through a Cu^2+^ oxide-filled 850°C quartz tube furnace, combusting unreacted ^14^CH_4_ to ^14^CO_2_. This ^14^CO_2_ was collected in two scintillation vials (23-ml volume) prefilled with 1 ml phenylethylamine and 7 ml 2-methoxyethanol, to which 10 ml of scintillation cocktail (Ultima Gold XR; PerkinElmer) was added. After a 24-h waiting period, radioactivity from ^14^CO_2_ was measured by scintillation counting (Beckman Coulter, Inc.; LS 6500 multipurpose scintillation counter, 10-min analysis per sample).

To quantify labeled ^14^C-labeled inorganic carbon produced during the incubation, the entire volume of each incubation sample was transferred into a 250-ml Erlenmeyer flask and 1 drop of antifoam was added. After a stopper was inserted, 5 ml of 6 M HCl was injected with a needle positioned along the side of the stopper. After injection, the needle was quickly removed and the flask was sealed with two clamps with Parafilm wrapping around the stopper to prevent gas escape. The flask was then placed on a shaking table (60 rpm, room temperature, 24 h). To collect ^14^CO_2_ generated by the acidification process, a 7-ml scintillation vial was prefilled with 1 ml of 2.5% NaOH and 1 ml of phenylethylamine and suspended from the rubber stopper inside the flask. After the shaking and acidification steps, 5 ml of scintillation cocktail was added, and the vial was measured by scintillation counting after 24 h. This method has been demonstrated to recover 98% of ^14^CO_2_ on average (37).

Finally, sterilized control samples (see Table S3) were set aside after ^14^CH_4_ addition to determine the initial concentration of methane gas. Four hundred microliters of headspace was injected into a gas chromatograph (Shimadzu GC-2014) equipped with a packed stainless steel Supelco custom column (50/50 mixture, 80/100 Porapak N support, 80/100 Porapak Q column, 6 ft by 1/8 in) and a flame ionization detector. The carrier gas was helium at a flow rate of 30 ml ⋅ min^−1^, and the column temperature was 60°C. Results were scaled based on comparison with standards of known methane concentrations (10 and 100 ppm; Matheson Tri-Gas, Twinsburg, OH). The rate of methane oxidation was determined by equation 8.

(8) methane oxidation=CO214×CH4(C14H4+C14O2)×volume×T

in which ^14^CH_4_ is the combusted unreacted radiolabeled methane, ^14^CO_2_ represents the quantity of acidified oxidation product, CH_4_ signifies the initial quantity of methane in the experiment (ensuring that any increase in unlabeled methane via methanogenesis will not contribute to the calculation), volume is the volume of initial sediment or carbonate rock, and *T* is the time over which the incubation was active.

### Isotopic analysis of methane in the headspace.

The methane headspace was analyzed via ^1^H-NMR spectroscopy using a Varian, Inc., 400-MHz spectrometer with a broadband auto-tune OneProbe. Three hundred microliters of headspace was passed through CDCl_3_ with a fine needle to absorb the methane. ^1^H-NMR spectra were acquired at 298 K without spinning, using a repetition rate of 10 s to ensure reliable quantification. The spectra were simulated with the iNMR 4.1.7 software for the determination of the fractional abundances of the ^12^CH_4_, ^12^CH_3_D, ^13^CH_4_, and ^13^CH_3_D isotopologues.
