# Supplementary material for: Monodeuterated Methane, an Isotopic Tool To Assess Biological Methane Metabolism Rates
Source: mSphere. 2017 Aug 23;2(4):e00309-17. doi: 10.1128/mSphereDirect.00309-17 (PMC5566838; doi:10.1128/mSphereDirect.00309-17)
Supplement: FIG S1 [file sph004172344sf2.pptx]

## Slide 1
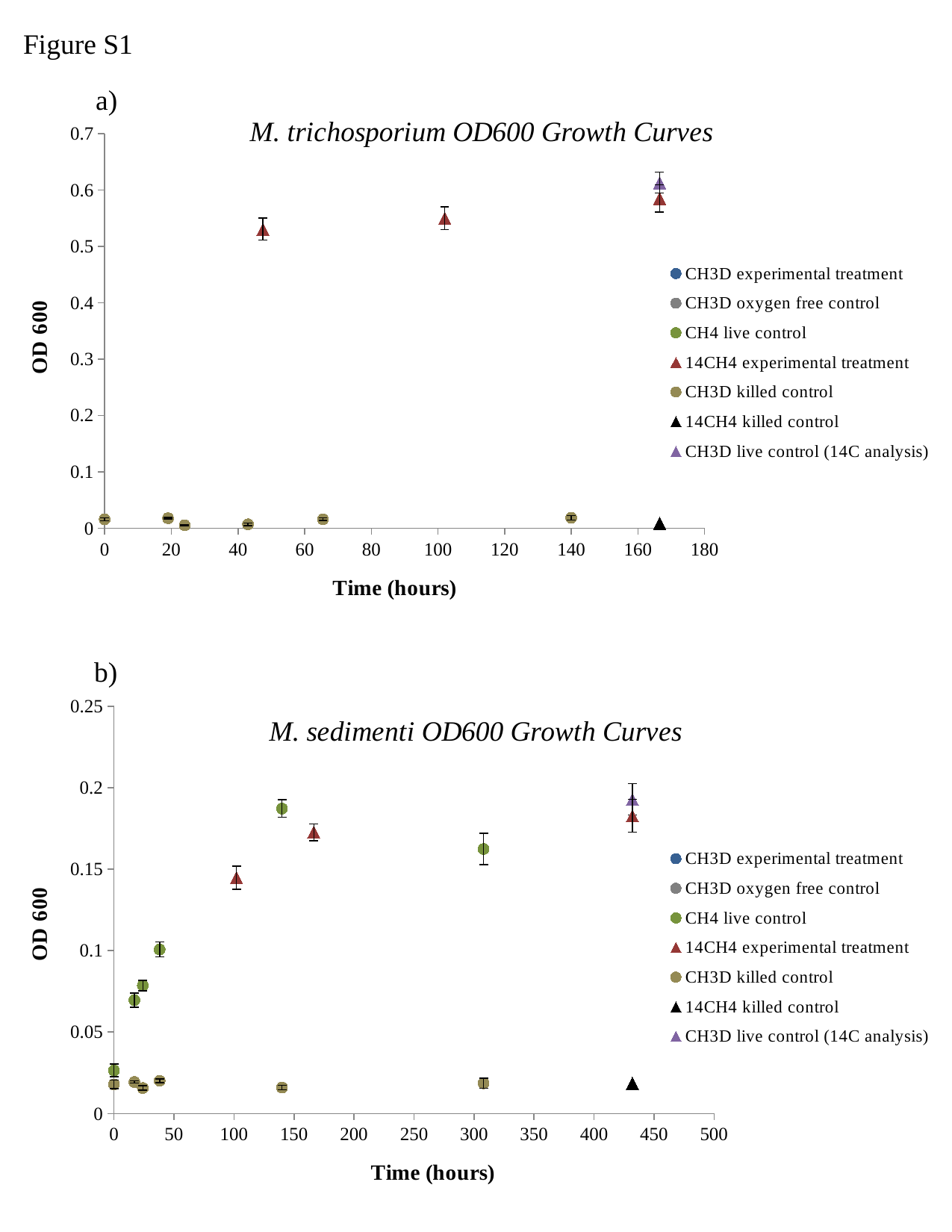

Figure S1
a)
### Chart: M. trichosporium OD600 Growth Curves
| Category | CH3D experimental treatment | CH3D oxygen free control | CH4 live control | 14CH4 experimental treatment | CH3D killed control | | |
|---|---|---|---|---|---|---|---|b)
### Chart: M. sedimenti OD600 Growth Curves
| Category | CH3D experimental treatment | CH3D oxygen free control | CH4 live control | 14CH4 experimental treatment | CH3D killed control | | |
|---|---|---|---|---|---|---|---|
