# Supplementary material for: Monodeuterated Methane, an Isotopic Tool To Assess Biological Methane Metabolism Rates
Source: mSphere. 2017 Aug 23;2(4):e00309-17. doi: 10.1128/mSphereDirect.00309-17 (PMC5566838; doi:10.1128/mSphereDirect.00309-17)
Supplement: TABLE S1 [file sph004172344st4.docx]

Table S1:

| **Sample #** | **Sediment (ml)** | **Nitrogen Source** | **Methane Source** | **Pressure (MPa)** |
| --- | --- | --- | --- | --- |
|  | | | | |
| 1a | 50 | 500 μM Glycine | 40 ml CH_3_D | 0.1 |
| 2a | 50 | 500 μM NH_4_Cl | 40 ml CH_3_D | 0.1 |
| 3a | 50 | 500 μM NH_4_Cl | 40 ml CH_4_ | 0.1 |
| 4a | 50, killed control | 500 μM Glycine | 40 ml CH_3_D | 0.1 |
|  | | | | |
| 1b | 50 | 500 μM Glycine | 40 ml CH_3_D | 9.0 |
| 2b | 50 | 500 μM NH_4_Cl | 40 ml CH_3_D | 9.0 |
| 3b | 50 | 500 μM NH_4_Cl | 40 ml CH_4_ | 9.0 |
| 4b | 50, killed control | 500 μM Glycine | 40 ml CH_3_D | 9.0 |
