# Supplementary figures and images for: Monodeuterated Methane, an Isotopic Tool To Assess Biological Methane Metabolism Rates
Source: mSphere. 2017 Aug 23;2(4):e00309-17. doi: 10.1128/mSphereDirect.00309-17 (PMC5566838; doi:10.1128/mSphereDirect.00309-17)

Table S2:


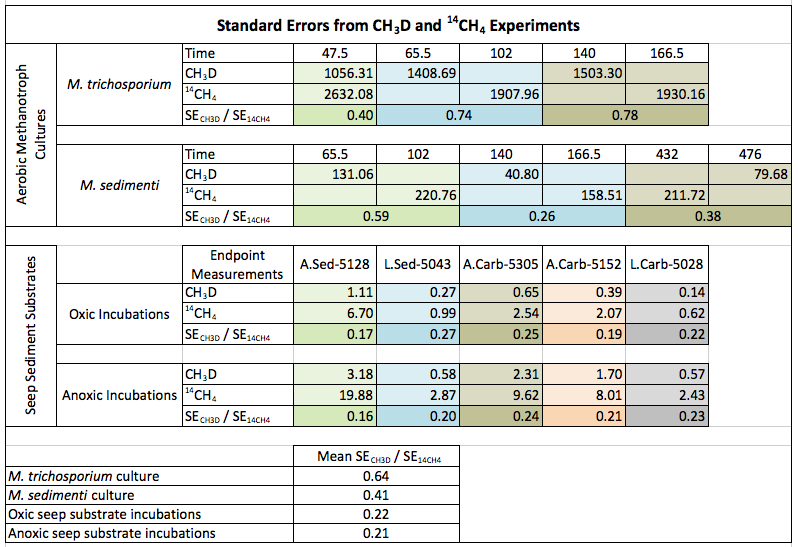

Supplement: TABLE S2 [file sph004172344st5.docx]

Table S4:


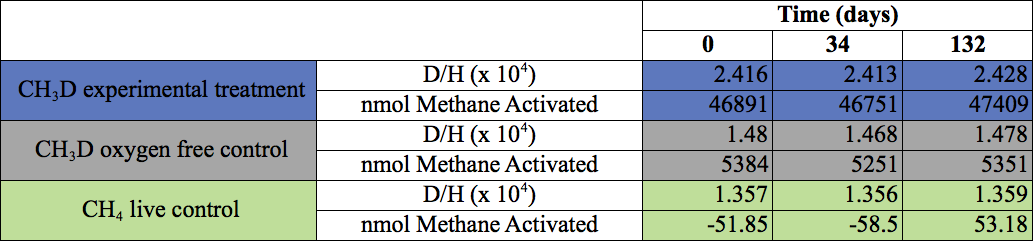

Supplement: TABLE S4 [file sph004172344st7.docx]
