# Supplementary material for: Monodeuterated Methane, an Isotopic Tool To Assess Biological Methane Metabolism Rates
Source: mSphere. 2017 Aug 23;2(4):e00309-17. doi: 10.1128/mSphereDirect.00309-17 (PMC5566838; doi:10.1128/mSphereDirect.00309-17)
Supplement: TABLE S3 [file sph004172344st6.docx]

Table S3:

| Sample Condition (Mode of Analysis) | [CH_3_D] mM | [CH_4_] mM | ^14^CH_4_ | [O_2_] mM | [Ar] mM | Inoculum (v/v) | Killed Cells |
| --- | --- | --- | --- | --- | --- | --- | --- |
|  | *Mt* // *Ms* | *Mt* // *Ms* | (kBq) | *Mt* // *Ms* | *Mt* // *Ms* |  |  |
| 1 (D/H_aq_) | 1 // 0.97 |  |  | 0.95 // 0.91 |  | 10% |  |
| 2 (D/H_aq_) | 1 // 0.97 |  |  | 0.95 // 0.91 |  | 10% | Yes |
| 3 (D/H_aq_) | 1 // 0.97 |  |  | 0.95 // 0.91 |  |  |  |
| 4 (D/H_aq_) | 1 // 0.97 |  |  |  | 1 // 0.98 | 10% |  |
| 5 (D/H_aq_) |  | 1 // 0.97 |  | 0.95 // 0.91 |  | 10% |  |
| 6 (^14^C) | 1 // 0.97 |  | 13 (T1) | 0.95 // 0.91 |  | 10% |  |
| 7 (^14^C) | 1 // 0.97 |  | 13 (T2) | 0.95 // 0.91 |  | 10% |  |
| 8 (^14^C) | 1 // 0.97 |  | 13 (T3) | 0.95 // 0.91 |  | 10% |  |
| 9 (^14^C) | 1 // 0.97 |  |  | 0.95 // 0.91 |  | 10% |  |
| 10 (^14^C) | 1 // 0.97 |  | 13 (T3) | 0.95 // 0.91 |  | 10% | Yes |
