# Supplementary material for: Monodeuterated Methane, an Isotopic Tool To Assess Biological Methane Metabolism Rates
Source: mSphere. 2017 Aug 23;2(4):e00309-17. doi: 10.1128/mSphereDirect.00309-17 (PMC5566838; doi:10.1128/mSphereDirect.00309-17)
Supplement: TEXT S1 [file sph004172344s1.docx]

Text S1

Resolution of the Monodeuterated Methane Measurement Approach

To determine the minimum number of activated CH_3_D molecules needed for analytical detection, we assessed the length of time required to measure a differentiable D/H ratio. Measurements were acquired at multiple time points between days 20 and 22 of a triplicate set of A.Sed-5128 incubations. A resolvable signal of an enhanced D/H ratio was defined as data points with non-overlapping confidence intervals, representing a 95% statistical probability that D/H ratios were increased. Such differentiation seen at the 20-hour sampling time for two replicates and the 26-hour sampling time for the other one (Fig. S2). Using the rate determined by the first 20 days as a baseline, this translates to a resolution of 4.5-6.2 μmol of fully oxidized methane based on the D:^14^C tracer ratio of 2.05 (Table 1).

Effect of Storage on D/H Values of Sampled Water

Storing water samples before analysis on the LWIA would be a helpful capability for users of the CH_3_D method: sample collection itself is rapid, which could lead to a backlog of samples awaiting analysis. Long-term storage would also enable rate-based experiments to be conducted – and water samples collected – in the field. To determine if storage of collected samples at 4 °C in GC vials for several months affected D/H ratios, we re-sampled water exhibiting a range of D/H values after 34 and 132 days. We used water samples from one replicate each of the CH_3_D experimental treatment, the CH_3_D oxygen-free control, and the CH_4_ live control from the 65.5-hour time point of the *M. trichosporium* aerobic methane oxidation experiment (Fig. 1a). Over the course of the next 132 days, D/H ratios changed by 0.5% (CH_3_D experimental treatment), 0.14% (CH_3_D oxygen-free control), and 0.15% (CH_4_ live control), indicating that storage of samples over several months is unlikely to compromise results (Table S4).

References

1. Weisenberger S, Schumpe A. 1996. Estimation of gas solubilities in salt solutions at temperatures from 273 K to 363 K. AIChE J 42:298–300.

2. Whittenbury R, Phillips K, Wilkinson J. 1970. Enrichment, isolation and some properties of methane-utilizing bacteria. Journal of General Microbiology 61:205–218.

3. Tavormina PL, Hatzenpichler R, McGlynn S, Chadwick G, Dawson KS, Connon SA, Orphan VJ. 2015. Methyloprofundus sedimenti gen. nov., sp. nov., an obligate methanotroph from ocean sediment belonging to the “deep sea-1”clade of marine methanotrophs. International journal of systematic and evolutionary microbiology 65:251–259.
