# Supplementary material for: Monodeuterated Methane, an Isotopic Tool To Assess Biological Methane Metabolism Rates
Source: mSphere. 2017 Aug 23;2(4):e00309-17. doi: 10.1128/mSphereDirect.00309-17 (PMC5566838; doi:10.1128/mSphereDirect.00309-17)
Supplement: FIG S2 [file sph004172344sf3.pptx]

## Slide 1
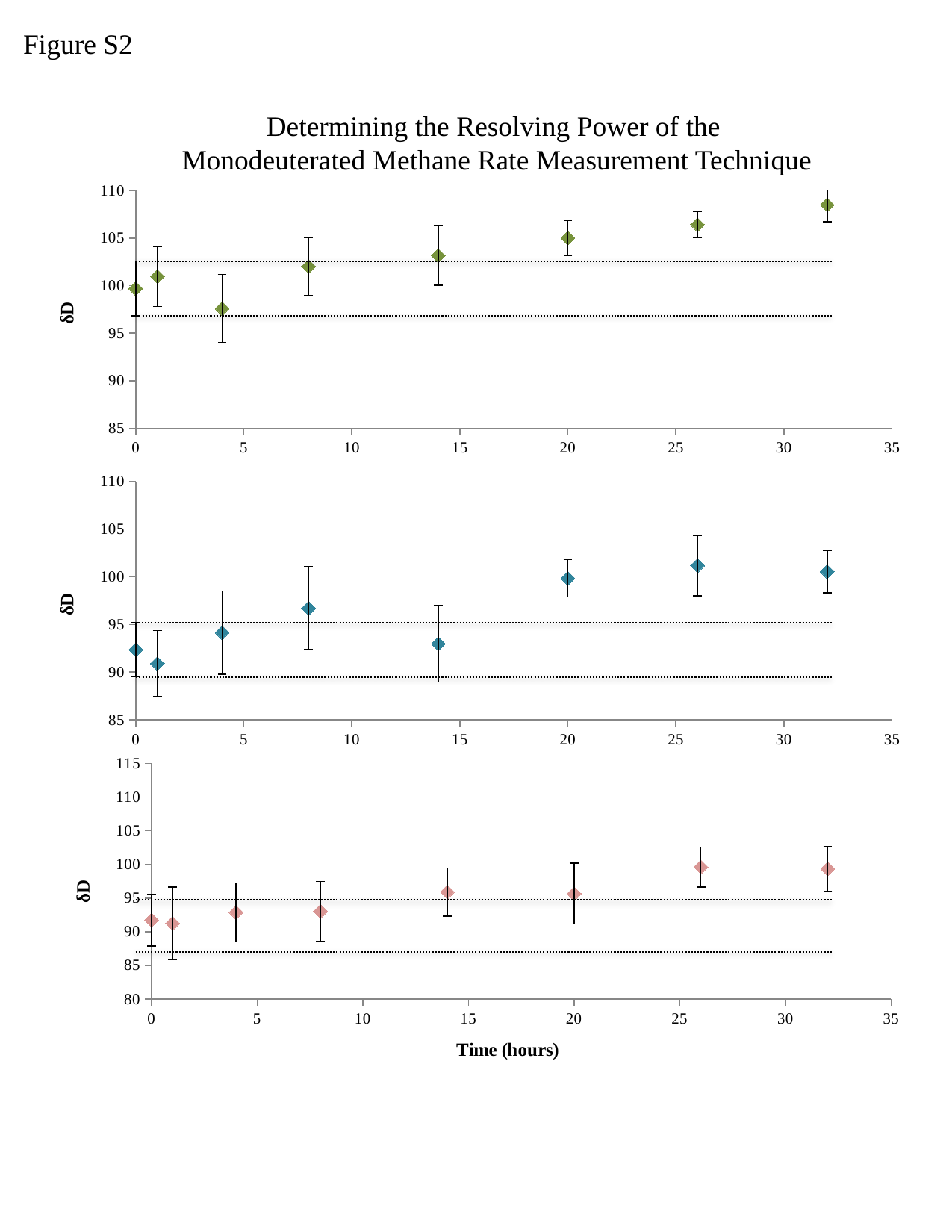

Figure S2
Determining the Resolving Power of the
Monodeuterated Methane Rate Measurement Technique
### Chart
| Category | |
|---|---|
### Chart
| Category | |
|---|---|
### Chart
| Category | |
|---|---|
